# Supplementary material for: Time-gated detection of protein-protein interactions with transcriptional readout
Source: eLife. 2017 Nov 30;6:e30233. doi: 10.7554/eLife.30233 (PMC5708895; doi:10.7554/eLife.30233)
Supplement: Transparent reporting form [file elife-30233-transrepform.docx]

***eLife’s* transparent reporting form**

We encourage authors to provide detailed information *within their submission* to facilitate the interpretation and replication of experiments. Authors can upload supporting documentation to indicate the use of appropriate reporting guidelines for health-related research (see [EQUATOR Network](http://www.equator-network.org/%20)), life science research (see the [BioSharing Information Resource](https://biosharing.org/)), or the [ARRIVE guidelines](http://www.plosbiology.org/article/info:doi/10.1371/journal.pbio.1000412) for reporting work involving animal research. Where applicable, authors should refer to any relevant reporting standards documents in this form.

If you have any questions, please consult our Journal Policies and/or contact us: [editorial@elifesciences.org](mailto:editorial@elifesciences.org).

**Sample-size estimation**

- You should state whether an appropriate sample size was computed when the study was being designed
- You should state the statistical method of sample size computation and any required assumptions
- If no explicit power analysis was used, you should describe how you decided what sample (replicate) size (number) to use

Please outline where this information can be found within the submission (e.g., sections or figure legends), or explain why this information doesn’t apply to your submission:

Our research does not require sample-size estimation.

**Replicates**

- You should report how often each experiment was performed
- You should include a definition of biological versus technical replication
- The data obtained should be provided and sufficient information should be provided to indicate the number of independent biological and/or technical replicates
- If you encountered any outliers, you should describe how these were handled
- Criteria for exclusion/inclusion of data should be clearly stated
- High-throughput sequence data should be uploaded before submission, with a private link for reviewers provided (these are available from both GEO and ArrayExpress)

Please outline where this information can be found within the submission (e.g., sections or figure legends), or explain why this information doesn’t apply to your submission:

In the context of the paper, a technical replicate refers to performing the assay multiple times using the same batch of transfected cells. Whereas, a biological replicate refers to the assay being performed using different batches of cells transfected with the same constructs.

Given the definition provided above, we wish to include the following information in the manuscript:

Figure 1C with three biological replicates. Figure 1D with two biological replicates (four technical replicates per biological replicate). Figure 1E with two biological replicates. Figure 1F with one biological replicate.

Figure 1-figure supplement 1A with two biological replicate (four technical replicates per biological replicate). Figure 1-figure supplement 1C-D with one biological replicate (four technical replicates). Figure 1-figure supplement 1E with one biological replicate (four technical replicates).

Figure 1-figure supplement 2A-C with one biological replicate. Figure 1-figure supplement 2D with two biological replicate (four technical replicates per biological replicate).

Figure 2B-C with one biological replicate. Figure 2D-E with one biological replicate (four technical replicates).

Figure 3C with one biological replicate (four technical replicates). Figure 3D-E with two biological replicates (four technical replicates per biological replicate).

Figure 4 with two biological replicates.

Figure 4-figure supplement 1B with one biological replicate (four technical replicates).

Figure 4-figure supplement 1C with two biological replicates (four technical replicates per biological replicate). Figure 4-figure supplement 1D and 1E with two biological replicates (four technical replicates per biological replicate).

No significant outliers encountered for any of the experiments described above.

**Statistical reporting**

- Statistical analysis methods should be described and justified
- Raw data should be presented in figures whenever informative to do so (typically when N per group is less than 10)
- For each experiment, you should identify the statistical tests used, exact values of N, definitions of center, methods of multiple test correction, and dispersion and precision measures (e.g., mean, median, SD, SEM, confidence intervals; and, for the major substantive results, a measure of effect size (e.g., Pearson's r, Cohen's d)
- Report exact p-values wherever possible alongside the summary statistics and 95% confidence intervals. These should be reported for all key questions and not only when the p-value is less than 0.05.

Please outline where this information can be found within the submission (e.g., sections or figure legends), or explain why this information doesn’t apply to your submission:

The only statistical analysis we performed is presented in line 184, which uses two sample Wilcoxon test (also known as the Mann-Whitney test) to test if the two variables have the same distributation. N=7 from two separate experiments.

(For large datasets, or papers with a very large number of statistical tests, you may upload a single table file with tests, Ns, etc., with reference to sections in the manuscript.)

**Group allocation**

- Indicate how samples were allocated into experimental groups (in the case of clinical studies, please specify allocation to treatment method); if randomization was used, please also state if restricted randomization was applied
- Indicate if masking was used during group allocation, data collection and/or data analysis

Please outline where this information can be found within the submission (e.g., sections or figure legends), or explain why this information doesn’t apply to your submission:

We did not use group allocation.

**Additional data files (“source data”)**

- We encourage you to upload relevant additional data files, such as numerical data that are represented as a graph in a figure, or as a summary table
- Where provided, these should be in the most useful format, and they can be uploaded as “Source data” files linked to a main figure or table
- Include model definition files including the full list of parameters used
- Include code used for data analysis (e.g., R, MatLab)
- Avoid stating that data files are “available upon request”

Please indicate the figures or tables for which source data files have been provided:

This does not apply to our submission, as we did not use source data files for any of the figures.
